# Modeling the Global Dynamic Contagion of COVID-19

**DOI:** 10.3389/fpubh.2021.809987

**Published:** 2022-01-14

**Authors:** Lijin Xiang, Shiqun Ma, Lu Yu, Wenhao Wang, Zhichao Yin

**Affiliations:** School of Finance, Shandong University of Finance and Economics, Jinan, China

**Keywords:** COVID-19 infections, time-varying connectedness, dynamic contagion, TVP-VAR model, spillover

## Abstract

The COVID-19 infections have profoundly and negatively impacted the whole world. Hence, we have modeled the dynamic spread of global COVID-19 infections with the connectedness approach based on the TVP-VAR model, using the data of confirmed COVID-19 cases during the period of March 23rd, 2020 to September 10th, 2021 in 18 countries. The results imply that, (i) the United States, the United Kingdom and Indonesia are global epidemic centers, among which the United States has the highest degree of the contagion of the COVID-19 infections, which is stable. South Korea, France and Italy are the main receiver of the contagion of the COVID-19 infections, and South Korea has been the most severely affected by the overseas epidemic; (ii) there is a negative correlation between the timeliness, effectiveness and mandatory nature of government policies and the risk of the associated countries COVID-19 epidemic affecting, as well as the magnitude of the net contagion of domestic COVID-19; (iii) the severity of domestic COVID-19 epidemics in the United States and Canada, Canada and Mexico, Indonesia and Canada is almost equivalent, especially for the United States, Canada and Mexico, whose domestic epidemics are with the same tendency; (iv) the COVID-19 epidemic has spread though not only the central divergence manner and chain mode of transmission, but also the way of feedback loop. Thus, more efforts should be made by the governments to enhance the pertinence and compulsion of their epidemic prevention policies and establish a systematic and efficient risk assessment mechanism for public health emergencies.

## Introduction

In December 2019, the COVID-19 epidemic merged in Wuhan, China, which has multiple characteristics, including high infectivity. The number of patients increased drastically at the beginning of the outbreak, due to the above characteristics of the epidemic (1). Within the following 2 months, the COVID-19 epidemic spreads to all provinces in China and all countries around the world, causing a serious global epidemic that has led to millions of confirmed and fatal cases worldwide, causing serious negative impacts on various economies and arousing widespread global attention. The WHO officially labeled the eruption of COVID-19 a pandemic on 11, March 2020.

The global spread of the COVID-19 epidemic not only poses a threat to life and property, but also a great challenge to global economic development, which increases the economic policy uncertainty (2, 3). In the meantime, the negative impact of COVID-19 will continue to be transmitted to the capital market through the industrial chain, the supply chain, and the capital chain, which will aggravate the volatility and risk contagion of domestic and foreign financial systems and severely impact the investing confidence of financial markets in various countries, increasing the possibility of an outbreak of global systemic financial risks and even a global economic crisis (4).

Although the infections of COVID-19 has been partially controlled through home quarantine, vaccination and other interventions in some countries; nonetheless, the transmission of COVID-19 is dynamic and repetitious, and most countries are still struggling to cope with repeated waves of epidemic caused by imported infections (5–10).

Modeling the global dynamic contagion of the COVID-19 infections is necessary to clarify the transnational transmission paths and control the contagion of overseas epidemic, so as to avoid frequent and repeated domestic epidemics. This study aims to examine and calculate the degree of the dynamic contagion of epidemic among countries and the transmission relevance, identify the time-varying and dynamic pattern of the epidemics contagion in sample countries. We hope the results could provide empirical evidence and insights for the policy-makers and scholars.

Considerable effort has been devoted to the study of the transmission dynamics of COVID-19. The current literature mostly focuses on using alternative data and models to dynamically predict the global spread of COVID-19, including predicting the fluctuation trend of the cumulative number of confirmed cases and the cumulative number of fatal cases, and measuring the R value.^1^ For example, Liu et al. (11) found that the average latent period of the COVID-19 epidemic was 4.8 days, and the methods of exponential growth (EG) and maximum likelihood (ML) were used to estimate the R value, with the results of 2.90 and 2.92, respectively. Zhu et al. (12) proposed a time delay reaction-diffusion model that is closer to the actual spread of the COVID-19 epidemic, taking into account the relapse, time delay, home quarantine and temporal-spatial heterogeneous environment that affect the spread of the COVID-19. The spread of the epidemic can be described more accurately by the main eigenvalue λ^2^ depicted in this model than the basic reproduction number-R value. In addition to the above models, SIR and SEIR models proposed by Kermack and McKendrick (13) have also been used to explore the spread of COVID-19. For example, Teles (14) simulated the MERS epidemic in Korea by using the SIR model to assess the evolution of the curve of the number of COVID-19 cases in Portugal. Tang et al. (15) derived the basic reproduction number of COVID-19 through SEIR model analysis. While Pandey et al. (16) employed the SEIR model and regression method to analyze and predict the development of the epidemic in India. Su et al. (17) adopted the SEIR model to estimate the basic reproductive number R0, which was 2.91 in Beijing, 2.78 in Shanghai, 2.02 in Guangzhou, and 1.75 in Shenzhen. Moreover, Huang et al. (18) proposed a time-dependent epidemic model, called T-SIR model, which could effectively model the epidemic dynamics of COVID-19 for all 50 states in the United States, providing insights into the transmission dynamics of the COVID-19 in that country. The Maximum-Hasting (MH) parameter estimation method and the SEIR model were used by Zhao et al. (19) to analyze the spread of COVID-19 in six African countries, which found that the spread of the epidemic depends on how local authorities intervened and what policies they adopted, and the same research was done by Yang et al. (20).

Subsequently, some scholars modified and advanced the SIR and SEIR models to predict the spread of the COVID-19 epidemic more accurately. For example, He et al. (21) applied a particle swarm algorithm to identify the parameters of the SEIR model and introduced seasonal and random infection parameters. Jiang et al. (22) made use of the SINDy-LM method—which balances complexity and prediction accuracy simultaneously—to simulate and research the COVID-19 transmission system in the Chinese mainland, Australia, and Egypt. Kamra et al. (23) applied the PolSIRD model to the spread of the COVID-19 outbreak in the United States; at the same time, counterfactual simulations from out model were also provided by them to analyze the effect of lifting the intervention policies prematurely. In addition, the second wave of the epidemic can correctly be predicted by the model. Based on the SIR and SEIR models, Zou et al. (24) proposed a new model, called the SuEIR model, that considers untested or unreported cases to analyze the spread of COVID-19. There is also a strand of literature that focuses on cluster analysis of the dynamic infectious characteristics of the COVID-19 epidemic in various countries around the world. For example, James and Menzies (25) proposed a cluster-based method to analyze the evolution of multivariate time series, which was applied to the COVID-19 epidemic by separating countries into clusters according to both their cases and death counts.

Furthermore, parts of the research models the global dynamic contagion of COVID-19 from the perspective of contagion networks. For example, Park (26) creates spatial visualizations of COVID-19 transmission network to study the spread of the COVID-19 in South Korea during the Early Epidemic Phase. Yum (27) employs social network analysis to explore how public key players play an important role in social networks for COVID-19. Jo et al. (28) create an infection network and analyze its structural characteristics, using contact tracing information of 3,283 confirmed patients in Seoul metropolitan areas from January 20, 2020 to July 19, 2020. The same method is also used by Wang et al. (29). Moreover, Skums et al. (30) exploited a network-based approach to analysis the transmission network of the SARS-CoV-2 epidemics before the pandemic declaration, employing molecular surveillance data of SARS-CoV-2 epidemics. Currently, the researches of the connectedness have been paid more attention by scholars, while the contagion of point-to-point transmission across countries based on the perspective of the global dynamic spreading structure of the COVID-19 epidemic has been little analyzed by the existing literatures. It is hard to provide a theoretical basis for the government to introduce targeted policies to prevent and control external COVID-19 epidemic impact by only predicting the global evolution trend of the epidemic and classifying and analyzing the epidemic characteristics of countries around the world based on SIR or SEIR and other alternative models. At the same time, conducting the social network analysis cannot clarify the degree of the COVID-19 contagion among countries and the pattern of cross-border epidemic transmission. While using the TVP-VAR-connectedness, not only can we depict the global transmission path of COVID-19 epidemic, but also clarify the level of the inter-country transmission correlation and the inter-country transmission tendency, which are the key to constructing an epidemic prevention and control system for each country and controlling the impact of external epidemic. Thus, inspired by the current literature, this paper uses the connectedness approach based on the TVP-VAR model to model the dynamic spread of the global COVID-19 epidemic.

The contributions of this paper are as follows:

From the perspective of the dynamic transmission structure of the COVID-19 epidemic, we depict the path of COVID-19's global contagion. We calculate the degree of the dynamic contagion of epidemic among countries to clarify the contagion capacity and relative severity of domestic epidemic and cross-border epidemic transmission pattern among countries around the world. We aim to deepen research on the dynamic transmission of the global COVID-19 epidemic, enrich the literature in this field and provide a theoretical and decision-making reference for countries to strengthen prevention and control of the transmission of overseas epidemic.

We combine the connectedness approach based on the TVP-VAR model with research on the dynamic transmission of the global COVID-19 epidemic. This approach builds on the work of Antonakakis et al. (31, 32) and Gabauer (33, 34) who advances the connectedness approach proposed by Diebold and Yilmaz (35–37) and overcomes the burden of the often arbitrarily chosen rolling-window-size that can lead to erratic or flattened parameters and loss of valuable observations, being able to examining the dynamic connectedness at lower frequencies and limited time-series data. This method that is mostly applied to research the correlation effects of one-to-many, many-to-one and one-to-one among variables (38–40, 44) is introduced by us to model the dynamic transmission of the COVID-19 epidemic among countries to broaden the research of the dynamic transmission of the global COVID-19 epidemic.

## Methodology and Data

### Research Design

To characterize the dynamic spread of the global COVID-19 epidemic, we make use of the connectedness based on the TVP-VAR Model to measure the static and dynamic contagion across countries, depict the transmission path of COVID-19 epidemic among countries, and capture the change trends of one-to-one correlation degree among countries. In the meanwhile, the contagion tendency of the COVID-19 epidemic in various countries is identified to clarify the key countries to ensure the pertinence and effectiveness of the epidemic prevention policies.

### Methodology

In this paper, based on the TVP-VAR model, the dynamic contagion indexes are constructed from two perspectives: the dynamic net pairwise connectedness and the dynamic directional connectedness.

#### The TVP-VAR Model

In particular, the TVP-VAR model can be written as follows:

(1) $$\begin{array}{r} Y_{t}=\beta_{t}Y_{t-1}+\varepsilon -t.........\varepsilon_{t}|F_{t-1}\sim \;N\left(0,S_{t}\right) \end{array}$$

(2) $$\begin{array}{r} \beta_{t}=\beta_{t-1}+\nu_{t}.........\nu_{t}|F_{t-1}\sim \;N\left(0,R_{t}\right) \end{array}$$

where *Y*_*t*_, *Y*_*t*−1_ represents *N* × 1 an dimensional vector, β_*t*_ is an *N* × *N*_*p*_ dimensional time-varying coefficient matrix and ε_*t*_ is an *N* × 1 dimensional error disturbance vector with a *N* × *N* time-varying variance-covariance matrix, *S*_*t*_. The parameters *β*_*t*_ depend on their own values *β*_*t*−1_ and on a *N*_*p*_ × *N*_*p*_ dimensional error matrix with a *N* × *N*_*p*_ variance-covariance matrix.

The time-varying coefficients and error covariances are used to estimate the generalized connectedness procedure based on generalized impulse response function (GIRF) and generalized forecast error variance decomposition (GFEVD) developed by Koop et al. (41) and Pesaran and Shin (42). To calculate the GIRF and GFEVD, we transform the VAR to its vector moving average (VMA) representation, the expression is as follows:

(3) $$\begin{array}{r} Y_{t}=\beta_{t}Y_{t-1}+\varepsilon_{t} \end{array}$$

(4) $$\begin{array}{r} Y_{t}=A_{t}\varepsilon_{t} \end{array}$$

(5) $$\begin{array}{r} A_{0,t}=I \end{array}$$

(6) $$\begin{array}{r} A_{i,t}=\beta_{1,t}A_{i-1,t}+\ldots +\beta_{p,t}A_{i-p,t} \end{array}$$

where $\beta_{t}={\left[\beta_{1,t},\beta_{2,t},\cdots \;,\beta_{p,t}\right]}^{\prime }$ and $A_{t}={\left[A_{1,t},A_{2,t},\cdots \;,A_{p,t}\right]}^{\prime }$. Hence, β_*i,t*_ and *A*_*i,t*_ are *N* × *N* dimensional parameter matrices.

The GIRFs represent the responses of all countries following a change of COVID-19 in country *i*. We compute the differences between a *J*-step-ahead forecast where once the COVID-19 epidemic of country *i* broke out and once where the COVID-19 epidemic of country *i* did not break out. The differences can be accounted for to measure the magnitude of the contagion of country *i*, which can be calculated by the following:

(7) $$\begin{array}{r} GIR_{t}\left(J,\delta_{j,t},F_{t-1}\right)=E\left(Y_{t+J}|\varepsilon_{j,t}=\delta_{j,t},F_{t-1}\right)-E\left(Y_{t+J}|F_{t-1}\right) \end{array}$$

(8) $$\begin{array}{r} \psi_{j,t}^{g}\left(J\right)=\frac{A_{J,t}S_{t}\varepsilon_{j,t}}{\sqrt{S_{jj,t}}}\frac{\delta_{j,t}}{\sqrt{S_{jj,t}}}.........\delta_{j,t}=\sqrt{S_{jj,t}} \end{array}$$

(9) $$\begin{array}{r} \psi_{j,t}^{g}\left(J\right)=S_{jj,t}^{-\frac{1}{2}}A_{J,t}S_{t}\varepsilon_{j,t} \end{array}$$

where *J* represents the forecast horizon, δ_*j,t*_ represents the selection vector with one on the *j*th position and zero otherwise, and *F*_*t*−1_ represents the information set until *t* − 1.

#### The Construction of the Dynamic Net Pairwise Connectedness Index

Subsequently, we compute the GFEVD, which can be interpreted as the variance share one country has on others. These shares are then normalized, so that each row sums up to one, meaning that all countries together explain 100% of the COVID-19 epidemic of country *i*. This is calculated as follows:

(10) $$\begin{array}{r} {\overset{\sim }{\phi }}_{ij,t}^{g}\left(J\right)=\frac{\sum_{t=1}^{J-1}\psi_{ij,t}^{2,g}}{\sum_{j=1}^{N}\sum_{t=1}^{J-1}\psi_{ij,t}^{2,g}} \end{array}$$

with $\sum_{j=1}^{N}{\overset{\sim }{\phi }}_{ij,t}^{N}\left(J\right)=1$ and $\sum_{i,j=1}^{N}{\overset{\sim }{\phi }}_{ij,t}^{N}\left(J\right)=N$. Using the GFEVD, we construct the pairwise countries contagion index of COVID-19 epidemic, including the mean level ($C_{i\to j,t}^{g}\left(J\right)$) of spread from country *i* to country *j*, which is calculated as follows:

(11) $$\begin{array}{r} C_{i\leftarrow j,t}^{g}\left(J\right)=\frac{\overset{\sim }{\phi_{ij,t}^{g}}\left(J\right)}{\sum_{i=1}^{N}\overset{\sim }{\phi_{ij,t}^{g}}\left(J\right)}*100 \end{array}$$

The mean level ($C_{i\leftarrow j,t}^{g}\left(J\right)$) of spread from country *j* to country *i* can be calculated as follows:

(12) $$\begin{array}{r} C_{i\leftarrow j,t}^{g}\left(J\right)=\frac{\overset{\sim }{\phi_{ij,t}^{g}}\left(J\right)}{\sum_{i=1}^{N}\overset{\sim }{\phi_{ij,t}^{g}}\left(J\right)}*100 \end{array}$$

We extracted Formulas (11) and (12), and defined the net pairwise countries contagion of the COVID-19 epidemic from country *i* to country *j* as the contagion of the COVID-19 epidemic from country *i* to country *j* minus the contagion of the COVID-19 epidemic from country *j* to country *i*. This is calculated as follows:

(13) $$\begin{array}{r} C_{i,t}^{g}=C_{i\to j,t}^{g}\left(J\right)-C_{i\leftarrow j,t}^{g}\left(J\right) \end{array}$$

#### The Construction of the Dynamic Directional Connectedness Index

Based on the above study, we can calculate the magnitude of the contagion effect of COVID-19 epidemic between a country and other sample countries, such as the COVID-19 contagion transmitted by country *i* to other sample countries (*TO*_*it*_Contagion). The expression is as follows:

(14) $$\begin{array}{r} TO_{it}=\overset{N}{\underset{j=1,j\neq i}{\sum }}C_{i\to j,t}^{g}\left(J\right) \end{array}$$

For the COVID-19 contagion received by country *i* from other sample countries (*FROM*_*it*_ Contagion), the expression is as follows:

(15) $$\begin{array}{r} FROM_{it}=\overset{N}{\underset{i=1,j\neq i}{\sum }}C_{i\leftarrow j,t}^{g}\left(J\right) \end{array}$$

According to Formulas (13) and (14), the Net Contagion denotes the difference between *TO*_*it*_Contagion and *FROM*_*it*_ Contagion, such as from Formula (16):

(16) $$\begin{array}{r} {NET}_{it}={TO}_{it}-FROM_{it} \end{array}$$

As shown in Formulas (13) and (16), if $C_{i,t}^{g}$ and *NET*_*it*_ are positive, it means that the impact of country *i* on other countries in the network is greater than the reaction of other countries in the network. On the contrary, if $C_{i,t}^{g}$ and *NET*_*it*_ are negative, country *i* is impacted by other countries in the network and accepts their net contagion.

### Data

As per the situation of COVID-19 epidemic around the world, we selected eighteen G20 countries in which EU and China are not included in are selected as sample with a wide range (see Table 1 for details), including developed countries and developing countries that locate in Asia, North and South America, Africa, Europe and Oceania. The sum of these countrie's GDP in 2020 accounts for 62.19% ^3^ of the total global GDP, the sample is representative.

**Table 1:** Sample countries and abbreviation.

**Country name**	**Abbreviation**
Argentina	AT
Australia	AU
Brazil	BR
Canada	CA
France	FR
German	GE
India	IN
Indonesia	ID
Italy	IT
Japan	JA
Korea	KO
Mexico	ME
Russian	RU
South Africa	SF
Turkey	TU
United States	US
United Kingdom	UK
Saudi Arabia	SA

In addition, the cross-border contagion effects of COVID-19 in different countries is expressed by the correlation of the spread speed of the COVID-19 epidemic in different economies, in which the spread speed is denoted by the first-order logarithmic difference of the number of confirmed COVID-19 cases. The cumulative data of the number of COVID-19 confirmed cases in various countries comes from the COVID-19 epidemic database of Johns Hopkins University.

The sample period starts at the peak of the global COVID-19 outbreak, and the time span covers the period from March 23, 2020, to September 10, 2021. Our weekly data contains a total of 1,386 observations from 18 selected countries.

## Empirical Results

### Static Analysis of Global Contagion of COVID-19 Infections

We use the TVP-VAR model to calculate the global cross-border contagion table of the COVID-19 epidemic. As shown in Table 2, the contagion of the COVID-19 epidemic of the sample countries is as high as 1600.3% overall, and the average cross-border contagion in a single economy is as high as 88.9%, which indicates that the COVID-19 epidemic has a significant contagion effect among countries. Of the sample countries, the United States, Canada, Mexico, Brazil, the United Kingdom, Indonesia, Russia and other countries have strong contagion on the system, while the net cross-border contagion of the COVID-19 epidemic in the United States is higher than other economies, reaching 19.9%, due to the fact that the United States has become a new COVID-19 epidemic center after China. For example, the number of newly confirmed COVID-19 cases in the United States has surged from March 2020, and domestic pessimistic expectations have been continuously enlarged under the economic impacted severely with panic spreading. For example, the stock market fused four times in an unprecedented way on March 9, 12, 16, and 18 2020. At the same time, the United States as the center of global economy has close economic and trade ties with many countries, which undoubtedly contributed to its being in a central contagion position within the COVID-19 epidemic. Additionally, the contagion of COVID-19 from the United States has also accelerated by the negative, anti-epidemic behavior of government authorities around the world.

**Table 2:** Cross-border contagion of COVID-19 epidemic.

	**AT**	**AU**	**BR**	**CA**	**FR**	**GE**	**IN**	**ID**	**IT**	**JA**	**KO**	**ME**	**RU**	**SF**	**TU**	**US**	**UK**	**SA**	**FROM**
AT	9.7	3.9	8.5	5.1	2.3	3.6	8.1	7.7	2.0	3.2	1.6	8.0	6.1	7.8	4.3	6.2	4.6	7.4	90.3
AU	5.9	14.4	4.2	6.7	2.9	6.3	4.0	5.2	4.4	5.1	1.9	4.7	4.2	6.1	7.0	8.1	6.2	3.0	85.6
BR	6.5	2.8	8.6	6.1	2.9	3.9	7.5	7.6	2.7	4.2	0.5	8.3	7.9	5.7	5.2	6.2	5.6	8.0	91.4
CA	4.9	5.0	5.8	8.1	3.8	6.2	5.1	6.2	4.9	5.4	0.6	6.1	6.7	4.2	7.3	7.3	7.4	5.0	91.9
FR	4.1	4.0	4.4	6.1	10.7	7.0	5.1	7.0	6.1	6.2	0.6	5.4	6.2	3.0	5.9	6.7	7.4	4.0	89.3
GE	4.2	5.1	4.0	7.7	4.4	10.0	3.6	5.2	8.0	4.2	2.4	4.4	5.2	3.5	8.3	8.0	8.2	3.6	90.0
IN	7.1	4.1	8.2	5.8	3.2	3.5	8.5	7.3	2.2	5.3	1.2	7.9	7.4	5.4	4.2	6.4	5.6	6.9	91.5
ID	6.7	3.4	7.3	5.9	3.9	4.4	7.1	8.0	2.9	4.9	0.8	7.5	7.1	5.5	5.5	6.6	5.8	6.8	92.1
IT	3.4	4.1	3.8	8.2	5.8	9.5	3.1	4.9	12.6	4.8	1.0	4.4	5.4	2.6	7.7	6.8	8.5	3.4	87.4
JA	4.6	6.8	5.3	6.8	4.1	5.5	6.0	6.3	3.8	9.6	1.0	5.7	6.5	2.9	7.0	7.2	6.6	4.4	90.4
KO	3.2	3.1	4.1	3.5	1.2	3.1	4.8	3.3	4.0	2.4	37.4	4.0	3.5	2.9	9.8	2.4	2.0	5.5	62.6
ME	6.5	3.3	8.2	5.9	3.3	3.5	7.5	7.7	2.4	5.1	0.4	8.4	7.8	5.8	4.8	6.2	5.5	7.7	91.6
RU	5.1	3.5	7.0	6.6	4.2	4.8	6.8	7.2	3.6	6.0	0.2	7.3	8.0	4.2	5.4	6.7	6.7	6.7	92.0
SF	8.4	4.7	8.6	4.8	2.1	2.8	7.1	7.7	1.5	3.2	1.2	8.9	6.5	10.3	4.0	5.9	4.3	8.3	89.7
TU	4.7	5.3	5.1	8.1	4.0	7.0	4.4	6.1	5.1	5.3	0.9	5.5	5.9	4.0	10.1	7.5	6.8	4.2	89.9
US	5.8	5.8	5.8	7.1	3.6	6.6	5.4	6.5	4.3	5.1	0.3	6.1	6.3	5.0	6.4	8.0	7.2	4.9	92.0
UK	4.6	4.5	5.2	7.5	4.5	7.6	4.8	6.2	5.7	5.2	0.4	5.7	6.7	3.9	6.5	7.8	8.6	4.8	91.4
SA	6.1	2.5	8.2	5.8	3.3	3.7	7.6	7.8	2.7	4.8	0.9	8.3	8.2	5.4	5.0	5.9	5.2	8.7	91.3
TO	91.7	71.8	103.3	107.4	59.3	89.1	97.9	109.8	66.3	80.3	15.7	108.1	107.6	77.9	104.0	111.9	103.5	94.6	1600.3
NET	1.4	−13.8	12.0	15.5	−30.0	−0.9	6.5	17.8	−21.2	−10.1	−47.0	16.6	15.7	−11.9	14.1	19.9	12.1	3.3	88.9

*The names of the economies in the table are expressed in abbreviations*.

The severity of the COVID-19 epidemic in some countries that are geographically adjacent or close to the United States has increased since the outbreak of COVID-19 in the United States; these countries include Mexico, Canada, and Brazil, amongst others, and their net contagion has also increased to 16.6, 15.5, and 12%. Hence, the geographical location of the three countries that provides a natural channel for the cross-border spread of the epidemic, and the degree of the contagion of COVID-19 epidemic among countries around the world is affected by the geographical distance.

As far as the major net receivers of COVID-19 contagion are concerned, South Korea, Japan and some European countries are at the forefront. For example, the contagion degree of South Korea from other economies is as high as 47%, while Japan's is 10.1% and those of France and Italy are 30 and 21.2%, respectively. The policies about compulsory shutdown and home quarantine were adopted earlier by the above-mentioned countries, which effectively inhibited the contagion of COVID-19, thus the difficulties of anti-epidemic in those countries come from the impact of COVID-19 from outside, which is why these countries present the characteristics of being affected by the COVID-19 epidemic of other countries.

**Figure 1:**
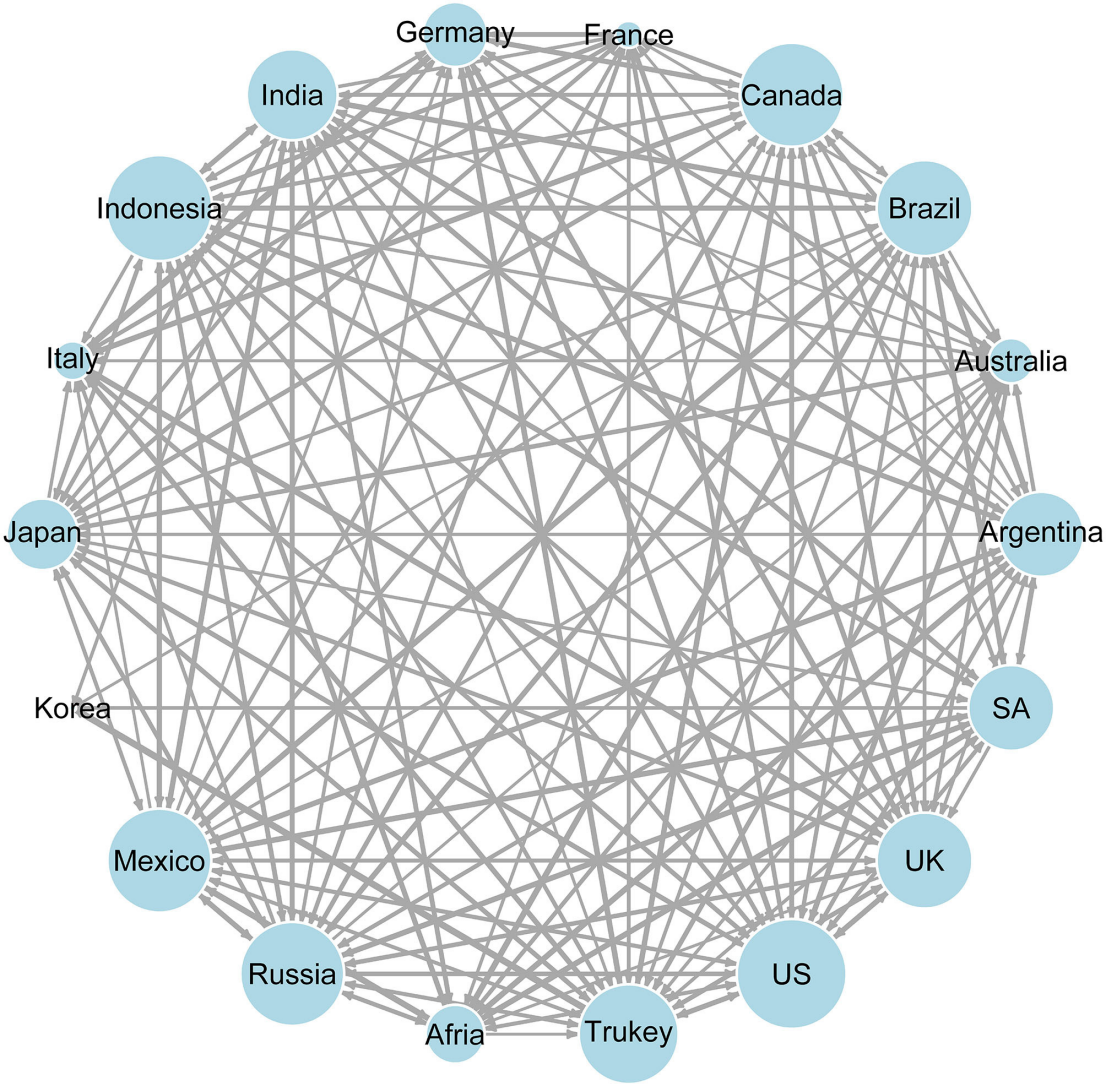
Directed weighted network for cross-border transmission of the COVID-19 epidemic. The contagion index between countries is taken as the edge weighting, and the contagion between countries is expressed by the thickness of the edge. Meanwhile, the total contagion of each country which is approximated by the size of the “To” index is indicated by the node size.

The contagion pattern among selected countries is presented in Figure 1. The network nodes for the United States, Canada, Mexico, Brazil, Turkey, Russia and the United Kingdom are large and prominent, while for South Korea, France, Italy and other countries, the opposite is true, which suggests that they are COVID-19 receiver of the contagion of the COVID-19 epidemic in other countries. Moreover, it can be seen from the edge thickness of the network topological graph that there is obvious heterogeneity in the contagion effects among countries. In particular, the conclusion is as mentioned above.

### Analysis of Global Dynamic Contagion of COVID-19 Infections

**Figure 2:**
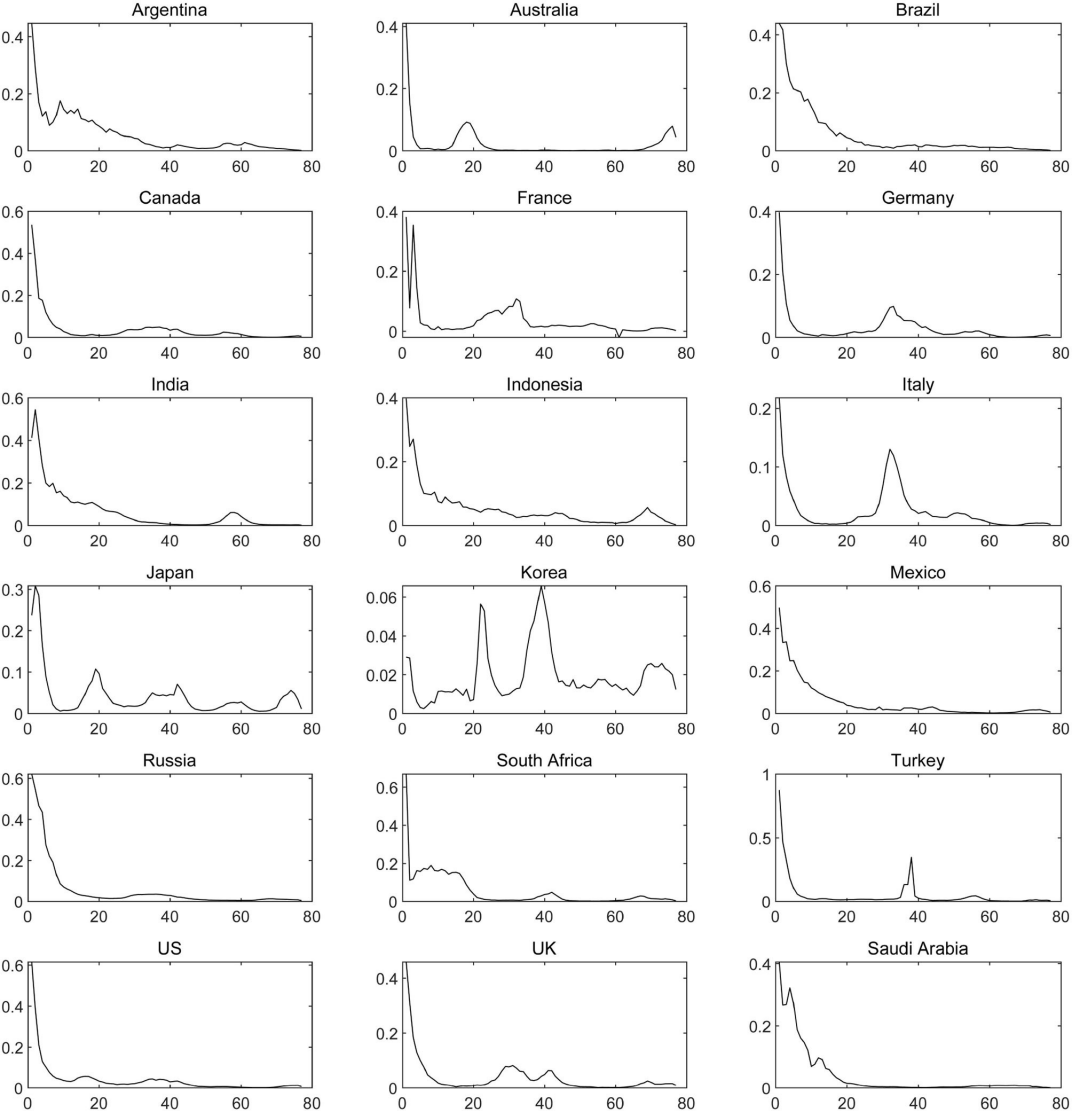
The growth rate of the number of confirmed cases of COVID-19 in various countries. The solid line represents the growth rate of the number of confirmed COVID-19 cases.

Dynamics analysis of the cross-border transmission of the COVID-19 among countries around the world is the key to simulating the dynamic transmission of COVID-19. Consequently, based on the first-order log-differential data of the number of confirmed COVID-19 cases in various countries, we analyze the trend of COVID-19 in different countries from a dynamic perspective, concluding that the time-varying characteristic can be reflected in the process of the transmission of the COVID-19 epidemic, which is consistent with the finding in Alarcon et al. (5). As shown in Figure 2, the COVID-19 epidemic outbreak intensified in various countries from March 2020 to May 2020, during which time the growth rate of the number of confirmed COVID-19 cases also reached its peak. Along with an increase in the number of confirmed patients in various countries, and the gradual enhancement of anti-epidemic awareness and the intensity and effectiveness of epidemic prevention policies, the growth rate of confirmed COVID-19 cases has declined. However, COVID-19 has recurred occasionally in various economies in the post-epidemic era, which is highly dynamic due to the impact of frequent cross-border transmissions of epidemics among countries. In Figure 2, the epidemic is shown to have made a comeback in European countries in November 2020, and the growth rate of the number of confirmed cases of COVID-19 rebounded to varying degrees in European countries like Italy, France, Turkey, German, and the United Kingdom. What's more, the number of confirmed COVID-19 patients in South Korea increased sharply in mid to-late August and mid-December, 2020, and the COVID-19 epidemic of Japan began to intensify at the same time due to the geographical proximity between Japan and South Korea.

#### FROM and TO Connectedness Indices

**Figure 3:**
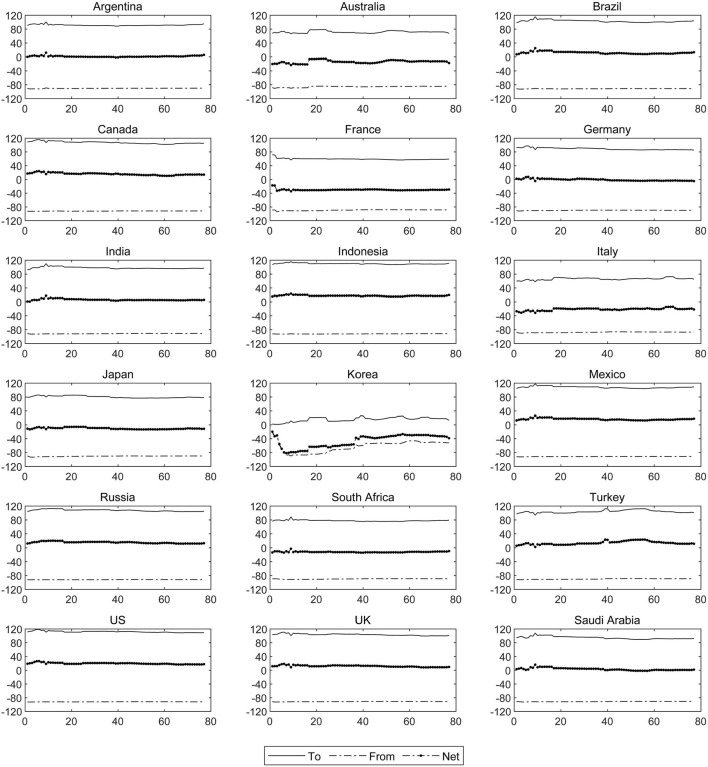
The dynamic directional connectedness of the COVID-19 epidemic. The solid line represents the “To Contagion” of COVID-19 epidemic in country *i* to other countries, while the dash line represents “From Contagion” of COVID-19 epidemic in country *i* by other countries, in which the directions of “To Contagion” and “From Contagion” are indicated by plus and minus sign respectively, while the dotted line represents the sum of the two, which is the “Net Contagion” of country *i*.

It is particularly necessary for clarifying the dynamic contagion status of each country to measure the dynamic directional contagion of COVID-19 in various countries around the world, clarify the characteristics of periodic changes of national net contagion, and then distinguish the sustainability of national COVID-19 cross-border contagion effects. This necessity arises from the fact that the repeated domestic epidemics in various countries have been caused by cross-border dynamic transmission of COVID-19. We measure the magnitude of the contagion effects of one country on other countries (To Contagion) and other countries on one country (From Contagion) and the net contagion of the sample countries, as shown in Figure 3.

The contagion roles played by most countries in the sample are stable to some extent; the United States, Canada, Mexico, the United Kingdom, Brazil, Russia, Indonesia, Turkey have strong contagion impact from a dynamic perspective. Additionally, the net contagion of each country is often negatively correlated with the effectivity, timeliness, and mandatory nature of the domestic anti-epidemic policies, especially under the trend of global economic integration. For example, the contagion of COVID-19 in the United States has been at a high level for a significant amount of time, and the reasons are as follows: first, the compulsory and urgent epidemic prevention of American government authorities who regard economic and employment data as political weights is insufficient, and partisan disputes are also an important obstacle to domestic anti-epidemic measures and the protection of people's right to life and health. Second, given the serious hollowing out of the American manufacturing industry, emergency reserve resources of medical supplies like respirators and masks are sufficient. As shown by the data provided by United States Health Secretary Azar, emergency reserves of medical supplies in the United States included only about 12 million medical-grade N95 masks and 30 million surgical masks, which has not been enough. Moreover, the partial privatization of medical institutions and medical insurance companies has led to high medical costs, which exacerbates the Matthew effect. Third, as the center of global economy, the United States maintains close economic and trade ties with other economies. For example, the United States is the core country in the North Atlantic Treaty Organization and a former member and leading country of the TPP Agreement, so the contagion of the United States' COVID-19 epidemic has been facilitated by the cross-border flow of resources, technology, and talents among these countries.

The levels of severity and external infectivity of the COVID-19 epidemic in Canada and Mexico are also high because the United States borders Canada to the north and Mexico to the south. Canada and Mexico's fluctuation trends of “From Contagion,” “To Contagion” and “Net Contagion” levels are particularly similar to those in the United States, and the tendency of the epidemic among the three countries is synchronized to some extent, which will necessitate higher requirements for epidemic prevention work in North America. In addition, Brazil also has a high magnitude of net contagion, which is closely related to the increasingly close economic and trade ties between Brazil and the United States. The United States has become Brazil's second largest trading partner since 2014, and a series of trade agreements has been signed by 2020, which has created a transmission link between the United States and Brazil, making the COVID-19 epidemic within the two countries resonate and form a negative cycle. Thus, Brazil's COVID-19 epidemic is spreading globally through the trend of economic globalization and population mobility globalization. At the same time, the frequent outbreaks of COVID-19 under the unfavorable domestic anti-epidemic situation in Brazil are also a critical reason for the country's high contagion level. The Indonesian government's slackness in preventing the epidemic and a lack of medical resources is also driving factors for the high cross-border contagion level under the continuous improvement of global economic integration.

Along with population mobility which aggravates the contagion and severity of domestic COVID-19 epidemics (43) such as overseas emigration, the contagion degree of COVID-19 in Turkey has been continuously increasing since 2021. Turkey is geographically bordered by Iran and Europe, and their close relationship due to the long-term economic development means that a large number of Turkish peoples work in Europe or Iran. Hence, population exchanges have intensified the directional contagion of COVID-19 between the countries involved. Especially in 2021, Turkey and other countries have successively resumed work and production, and their vigilance against COVID-19 and awareness of epidemic prevention has also relaxed, which has led to the acceleration of cross-border transmission of the epidemic in these countries. The direction of the net contagion of COVID-19 German changed at the beginning of December, 2020. This change was due to ever-increasing anti-epidemic measures, the epidemic prevention efficiency of the government, and the rich medical resources in the country, which ensured gradual control of the domestic COVID-19 epidemic. However, the overseas COVID-19 epidemics contagion is one of the reasons for periodic changes in the growth rate of the number of confirmed cases in German.

There are still some countries in the sample—such as South Korea, Italy and France—that have always been affected by COVID-19 epidemics in other countries. Among them, the country most significantly affected by the overseas epidemic is South Korea, which has actively adopted China's recommendations for confronting the COVID-19 pandemic, adopting plans for detection, tracking, isolation, and treatment, and frequently introducing policies to combat the epidemic. However, the effectiveness of domestic epidemic prevention and control has been seriously weakened by domestic party disputes. Moreover, religious groups have spread infections and refused to cooperate, continuously amplifying the speed of cross-border transmission and diffusion risk of overseas epidemics. In the meantime, complex and fragile domestic epidemic prevention environment, which include domestic employment pressure and frequent anti-COVID-19 vaccine activities, have also resulted in Italy and France becoming receiver of the contagion of the overseas epidemic.

#### Net Pairwise Directional Connectedness

The dynamic circumstances of the COVID-19 epidemic in various countries can be fully reflected via the measurement of the dynamic contagion of the COVID-19 epidemic in sample countries, which can also depict the role and its dynamic changes of each country's epidemic in time dimension. Moreover, it is meaningful to further analyze the micro-structure of COVID-19 cross-border contagion in various countries based on research on infection from one country to other countries and from other countries to one country, to clarify the cross-border contagion path. Thus, we will capture and identify the microscopic structure of the COVID-19 epidemic among countries, and then clarify the dynamic characteristics of the contagion.

**Figure 4:**
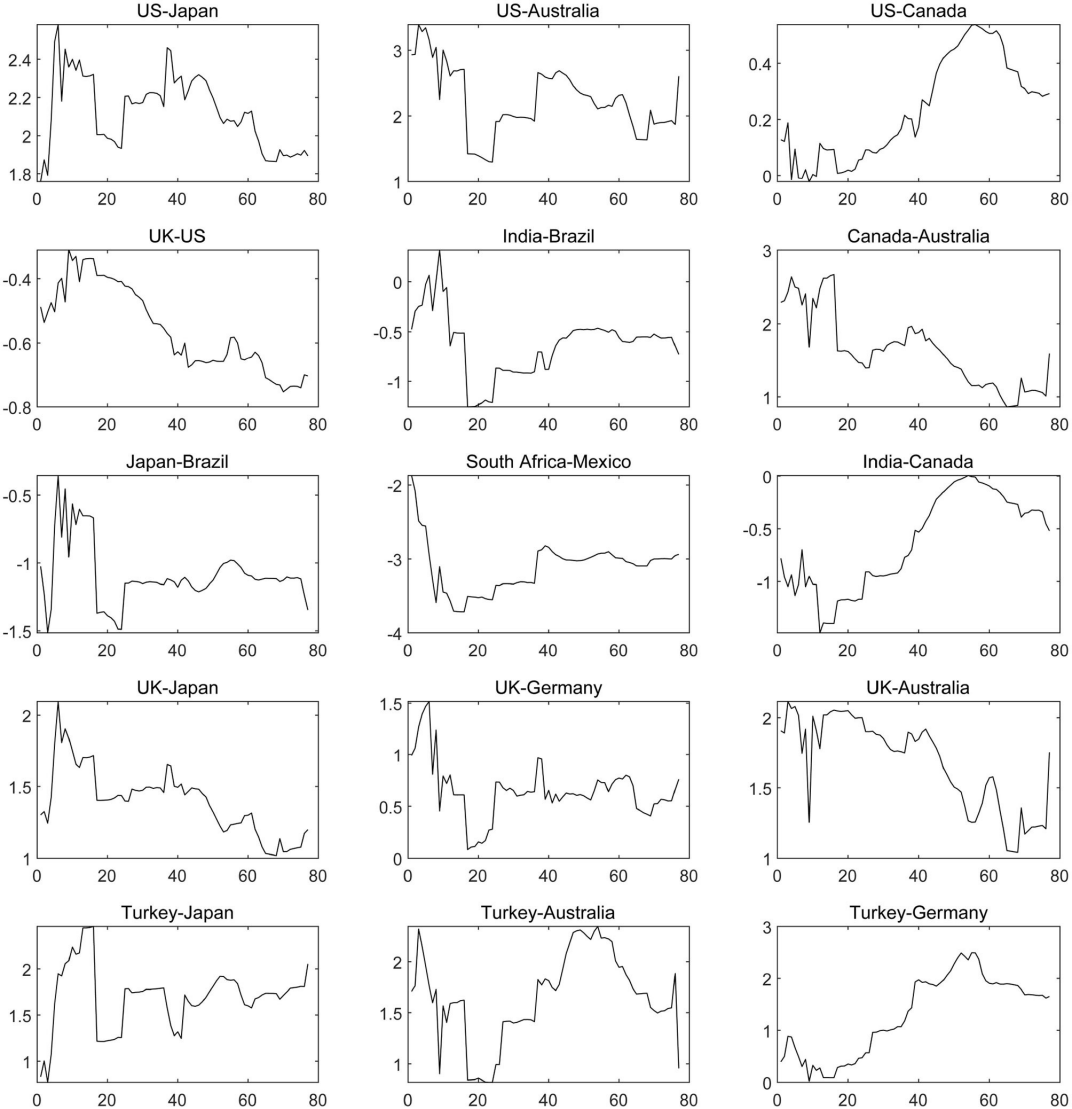
The dynamic net pairwise connectedness of the COVID-19 epidemic (Part one). The results are based on the connectedness approach based on TVP-VAR model, and the solid line represents the change trend of the net COVID-19 contagion from one country to another.

We depict the net contagion of the COVID-19 epidemic among sample countries. The results shown in Figure 4, suggest that the United States still maintains net contagion to all countries. In particular, the United States, Australia and Japan belong to the “Group of Four;” hence, close economic and trade contacts and frequent personnel mobility between all three countries allowed the access that the COVID-19 epidemic in the United States to spill over to Japan and Australia at an early stage. Given the adjacent geographical location of the United States and Canada, the COVID-19 epidemics in the two countries have a strong synchronization and correlation. When the United State's COVID-19 epidemic was still at its peak at the end of 2020, Canada's epidemic prevention began to decline; for example, family immigrants became largely exempted, and international students were allowed to enter the country. Thus, the contagion of COVID-19 from the United States on Canada has been constantly strengthening. In addition, the United States and the United Kingdom cooperate closely in the fields of trade, finance, science and technology, academia, art and military affairs. Additionally, the United States and the United Kingdom are important investors for each other, which has provided a channel for the contagion of COVID-19 from the United States to the United Kingdom. Countries near the United States geographically also transmitCOVID-19, such as Canada, Mexico and Brazil, which directionally affect India, Australia, Japan, South Africa and other countries, accelerating the global spread of the epidemic.

Europe is another main source of COVID-19 outbreaks, and the United Kingdom and Turkey are the main disseminators, and the United Kingdom has higher contagion level than Turkey. Both countries have persistent contagion on Japan, Australia and German, and the contagion effect of the epidemic between Turkey and German is increasing. The main reason for this increase is that there are a large number of Turkish immigrants in German, with close population mobility between the two countries, and the increase of population mobility after October 2020 under the staged relaxation of anti-epidemic measures has been a fuse for intensifying the transnational spread of COVID-19.

**Figure 5:**
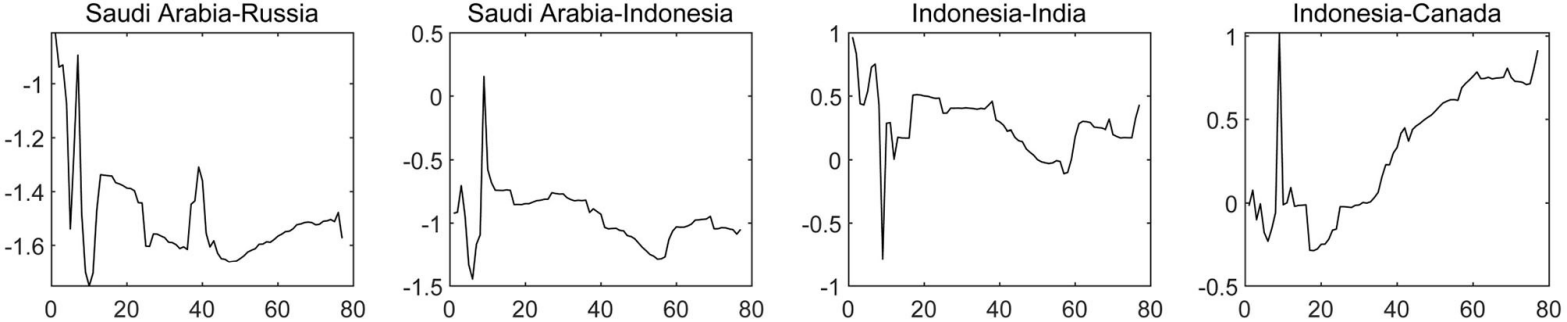
The dynamic net pairwise connectedness of the COVID-19 epidemic (Part Two). The results are based on the connectedness approach based on TVP-VAR model, and the solid line represents the change trend of the net COVID-19 contagion from one country to another.

As far as Asian countries and African countries are concerned, Russia and Indonesia whose spread paths have global characteristics are at a high level of net contagion. For example, South Africa, India and Canada can be affected across borders by the COVID-19 of Indonesia, which regards tourism that provides the conditions for the early global diffusion of domestic COVID-19 as the pillar industry of its domestic economy, as shown in Figure 5. Subsequently, many factors such as long-term shortage of medical materials (including vaccines), virus variants, a lack of long-term awareness of domestic epidemic prevention and weak mandatory bans led to the second outbreak of COVID-19 in Indonesia in 2021, and the contagion of COVID-19 from Indonesia to other economies increased again, such as to Canada and India. Consequently, the transmission of the global COVID-19 epidemic has been centered on the United States, the United Kingdom and Indonesia.

**Figure 6:**
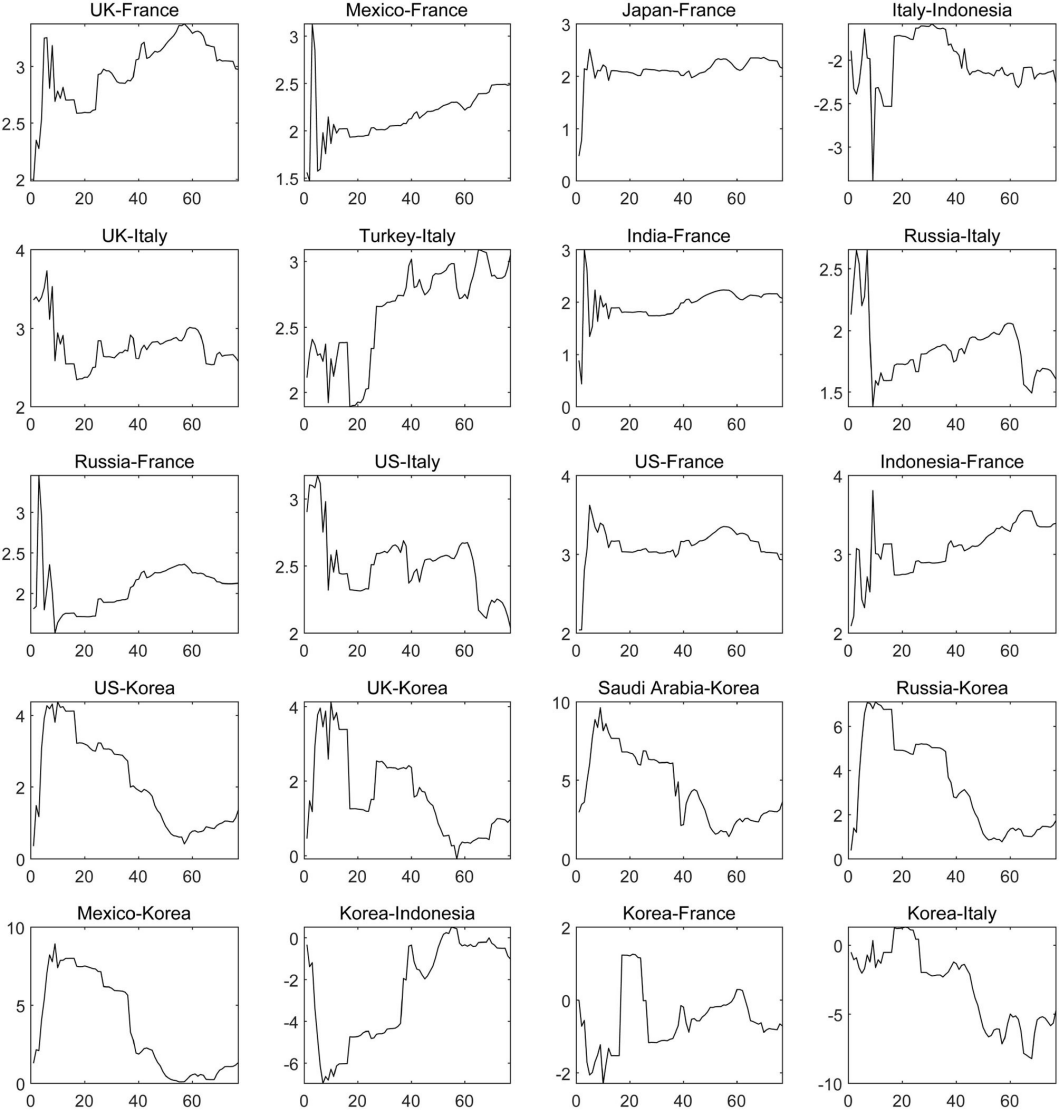
The dynamic net pairwise connectedness of the COVID-19 epidemic (Part Three). The results are based on the connectedness approach based on TVP-VAR model, and the solid line represents the change trend of the net COVID-19 contagion from one country to another.

Based on the sample countries, South Korea, France and Italy are intensively affected by the COVID-19 of countries around the world, as shown in Figure 6. Among them, in April 2020, the growth rate of domestic confirmed cases and the degree of being affected by overseas COVID-19 epidemics reached a peak in South Korea, which was related to the strong economic relationship between South Korea and other countries under the trend of global economic integration. For example, a free trade and cooperation agreement covering the fields of economy, trade, science and technology, health and education, has been signed between South Korea and other countries including Canada, countries in the EU and Brazil. Along with continuously strengthening multilateral economic and trade relations, the foreign direct investment flowing into South Korea has increased sharply. At the same time, South Korea, which is a major market in Asia, is also a major consumer of energy and products from chemical industries, which is attractive to foreign capital and talents and provides an opportunity and economic channel for transnational contagion of COVID-19. Since 2021, the net contagion of COVID-19 in South Korea from other countries, such as the United States, the United Kingdom, Russia, Mexico and other countries, has been declining, which is mainly due to the anti-epidemic level being raised to level 3, the strength of entry restrictions for overseas people and compulsory vaccination, indicating that enhancing mandatory and effective anti-epidemic policies can effectively reduce the risk of overseas epidemic affecting. As far as France and Italy are concerned, they have been impacted by contagion of the COVID-19 from the United States, the United Kingdom, Canada, Mexico, Turkey and Russia for a significant amount of time with stability.

Regarding the three major COVID-19 receiver—which are South Korea, France and Italy—South Korea still maintains the net receiver of the epidemic identity, which indicates that strengthening the intensity of prevention of abroad epidemic affecting is the top priority for South Korea, in its attempt to effectively control the domestic COVID-19 epidemic situation.

**Figure 7:**
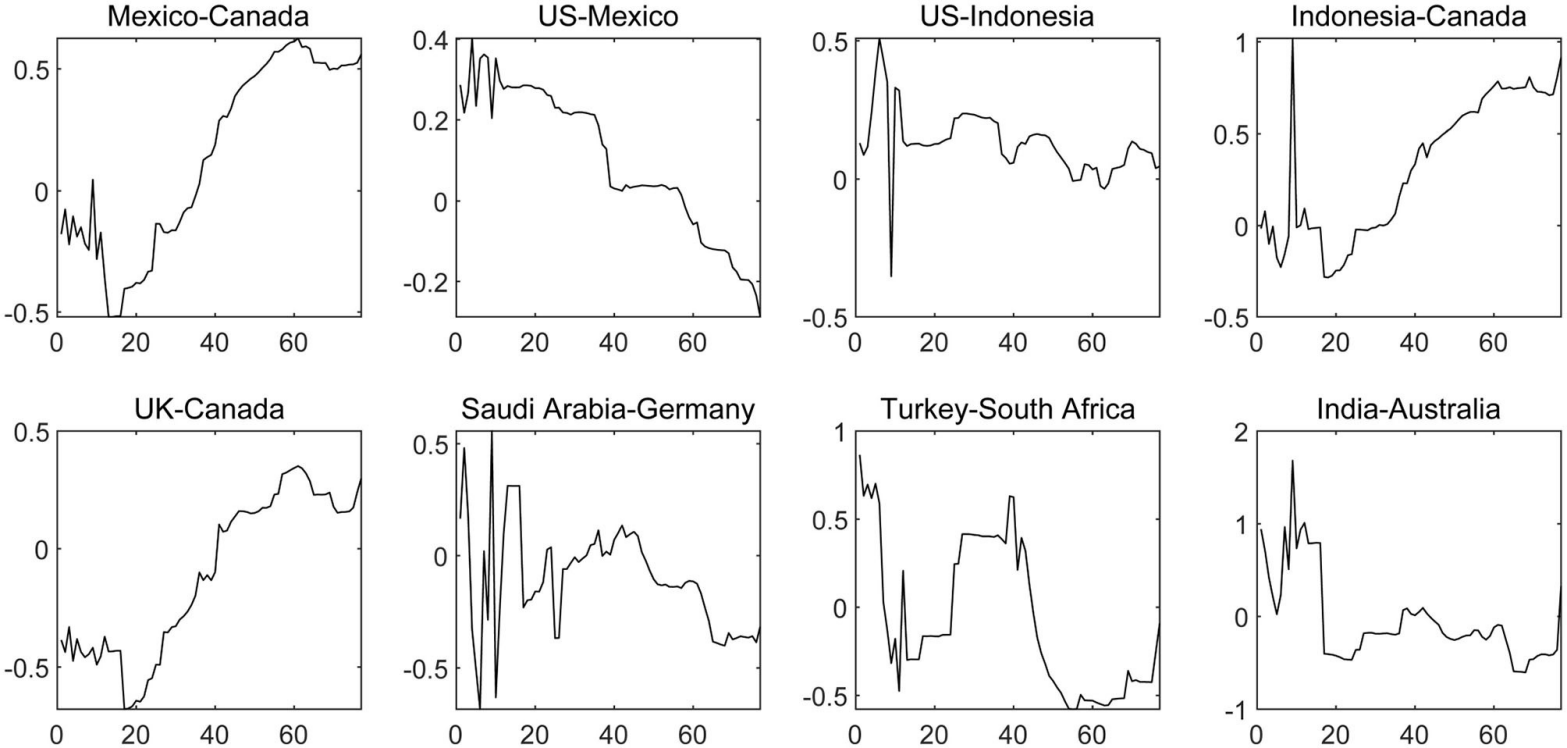
The dynamic net pairwise connectedness of the COVID-19 epidemic (Part Four). The results are based on the connectedness approach based on TVP-VAR model, and the solid line represents the change trend of the net COVID-19 contagion from one country to another.

There are still some countries with low net contagion level and in which the contagion direction has changed repeatedly during the sample period due to the slight dynamic change of COVID-19, such as Mexico and Canada, the United States and Canada, the United States and Indonesia, Indonesia and Canada, the United Kingdom and Canada and so on, as displayed in Figure 7. The above characteristics suggest that the severity of the COVID-19 epidemic is equivalent in each set of countries, which confirms the conclusions that the spread of COVID-19 in the United States, Canada and Mexico has the same trend and Indonesia has become a new global COVID-19 epidemic center.

### Cross-Border Contagion Tendency of the COVID-19 Epidemic

This paper is based on research on the micro-structure of the dynamic spread of COVID-19 epidemic among countries around the world. According to the dynamic contagion of COVID-19 between two countries, we rank the main “From” countries and “To” countries of the single economy, recording the top three countries, which effectively depict the tendency of domestic COVID-19 impact in sample countries. This depiction provides theoretical basis for countries to prevent the contagion of COVID-19 from overseas, control the overflow impact of domestic epidemics, and provide a decision-making reference for the government to issue relevant policies.

**Figure 8:**
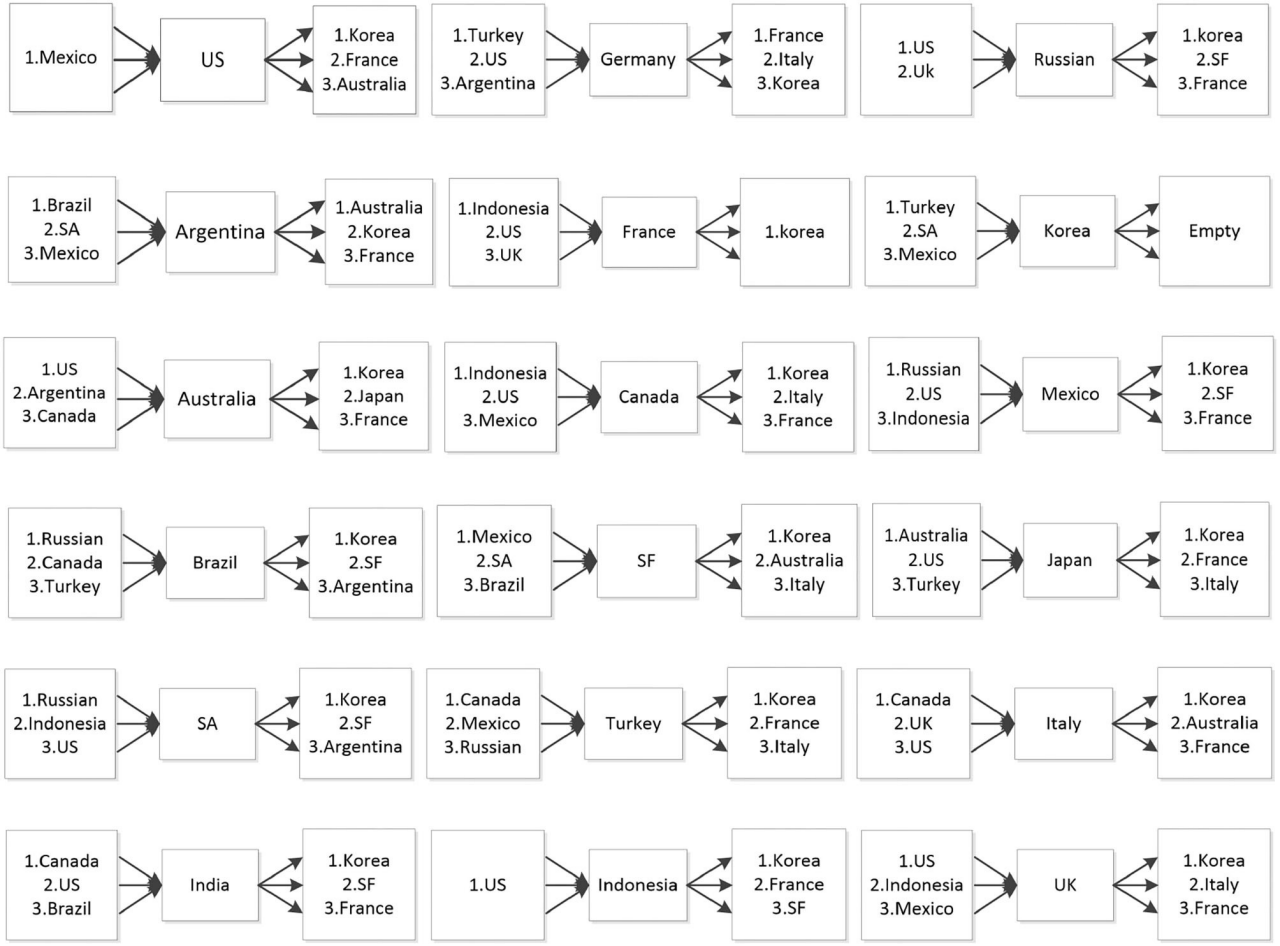
The main “To Contagion” countries and “From Contagion” countries of COVID-19.

In Figure 8, it is evident that COVID-19 epidemic centers at United States, the United Kingdom, Indonesia, Canada and Mexico among other sample countries, while South Korea, France and Italy are still receivers of the epidemic.

**Figure 9:**
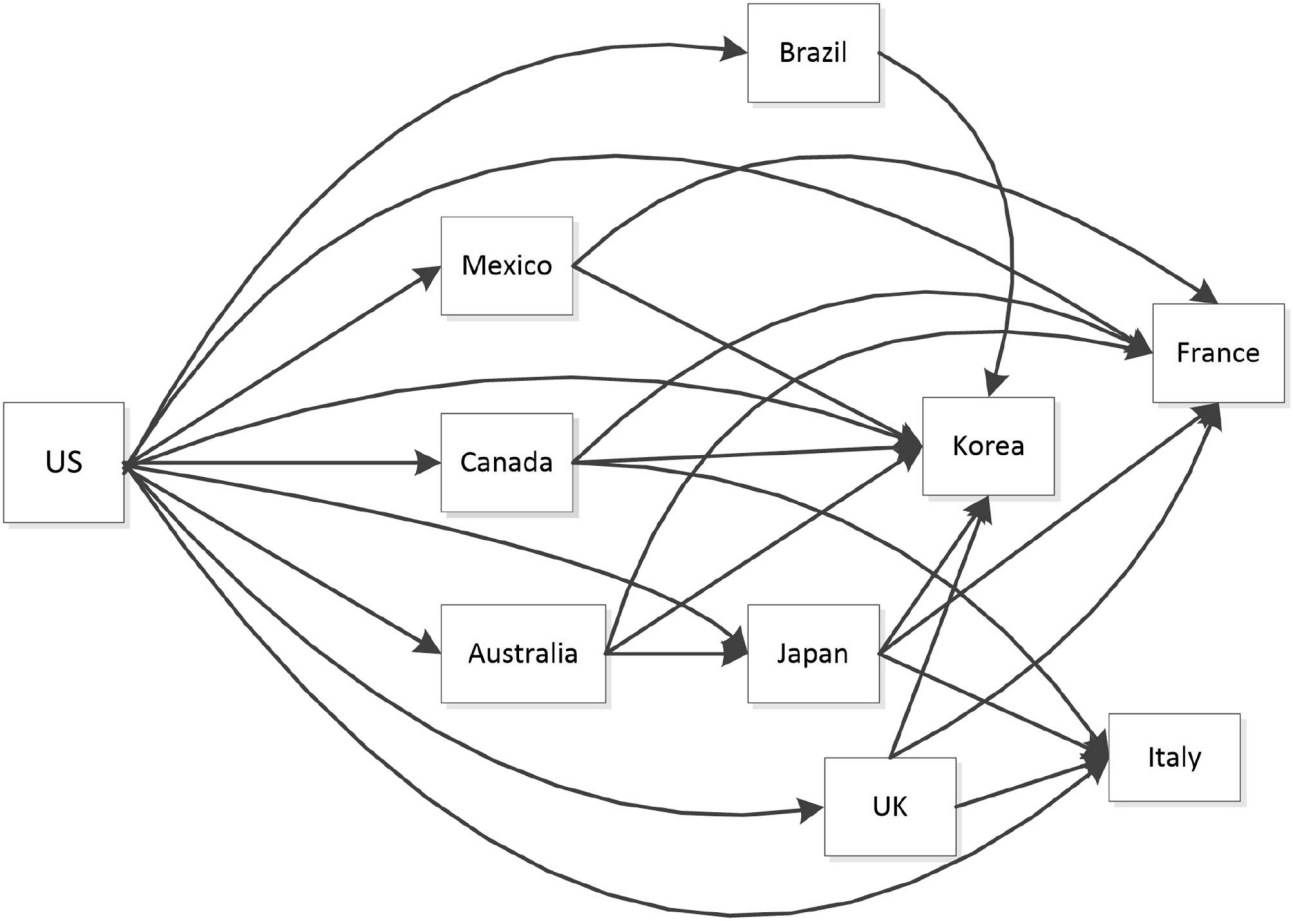
Paths of COVID-19 contagion from the United States.

**Figure 10:**
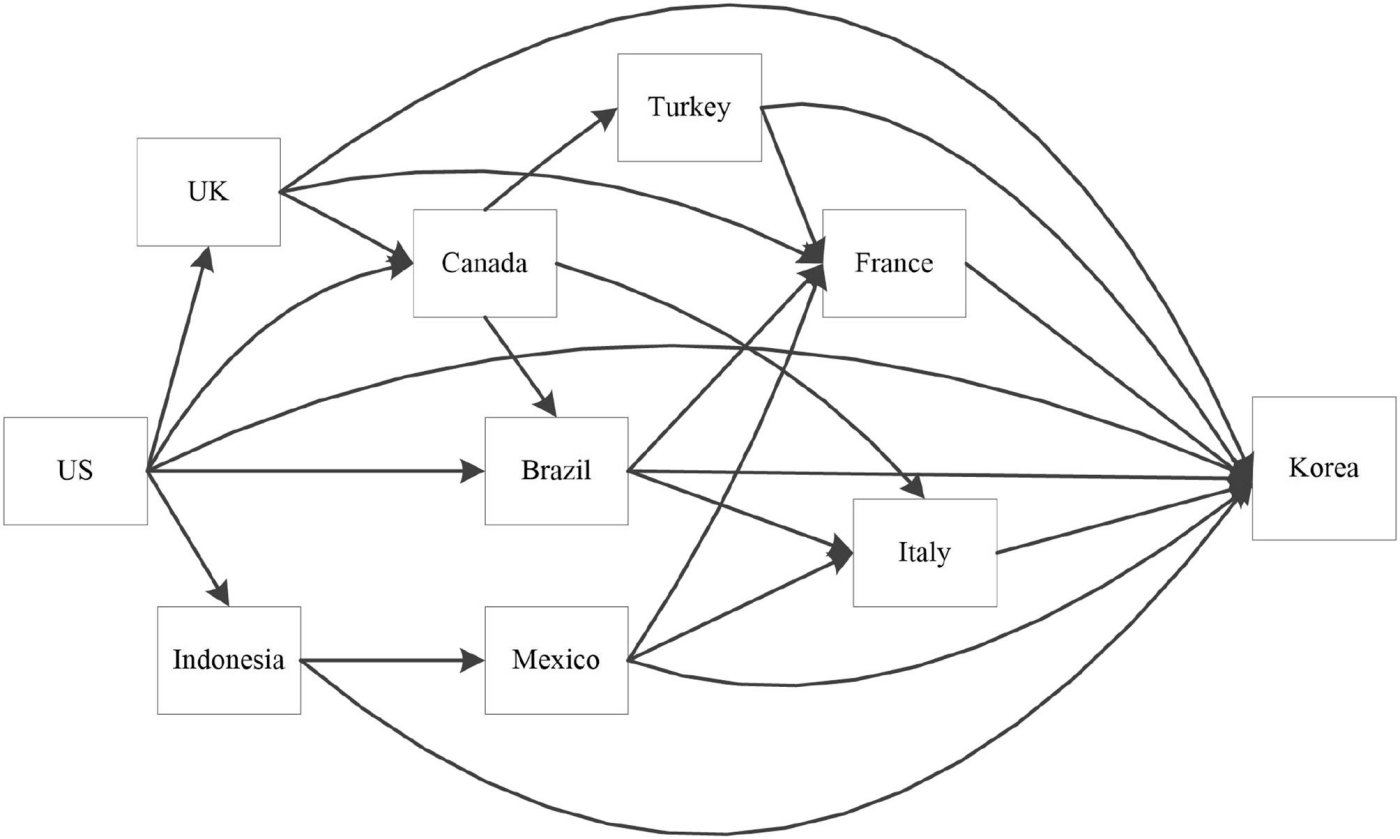
Paths of COVID-19 contagion to South Korea.

The COVID-19 epidemic not only spreads directly in a central divergent way, but also transmits indirectly through a complex network. For example, in the United States, as shown in Figure 9, the COVID-19 epidemic erupted in March 2020, diverging to Canada, Mexico, Australia, Japan, South Korea and other countries. And there are inter-transmission among these countries. As refer to the typical receiver, South Korea, the situation is another way around. The spillover of COVID-19 epidemic is represented in Figure 10.

At present, the major transmitters of the contagion of the COVID-19 epidemic including the United States, the United Kingdom and Indonesia should not only strengthen their domestic epidemic prevention, but also increase their efforts to control cross-border transmission of their own epidemics, especially for those countries with the deepest epidemic correlations, such as South Korea, France, Italy, Australia and South Africa. As for the major receivers of COVID-19, purposefully controlling overseas epidemic input and temporarily weakening the associations with sources countries of the epidemic are crucial prerequisites to avoid the frequent and repeated outbreak of domestic epidemics and to ensure macroeconomic stabilization and recovery.

## Conclusion

In this article, the connectedness approach based on the TVP-VAR model is used to construct a dynamic contagion index to model the global dynamic spread of COVID-19. The main conclusions of this paper are as follows:

First, the United States, the United Kingdom and Indonesia are the global epidemic centers, among which the United States has the highest level of COVID-19 epidemic contagion with contagion stability. Canada and Mexico who are geographically close to the United States, also have strong cross-border infectivity, and all three countries demonstrate the same development tendencies for their domestic epidemics, which necessitates higher requirements for anti-epidemic work in North America. South Korea, France and Italy are the major receivers of COVID-19 around the world, among which South Korea is the most seriously affected by overseas epidemics. Additionally, the promotion of compulsory, timely and effective anti-epidemic policies can impose a negative effect on the input from foreign COVID-19 epidemics, weakening their contagion of domestic epidemics.

Second, the severity of domestic COVID-19 epidemics in some countries is in an equal position according to the results. These countries include Mexico and Canada, the United States and Mexico, the United States and Canada, the United States and Indonesia, Indonesia and Canada, the United Kingdom and Canada, Turkey and South Africa, Saudi Arabia and German and so on, which means that the conclusion that the United States, the United Kingdom and Indonesia are the centers of the global COVID-19 epidemic is robust.

Third, the global COVID-19 pandemic centering on the United States, the United Kingdom and Indonesia, not only spreads directly from near center to far, but also transmits indirectly through chain unidirectional conduction.

Some policy implications can be drawn from the above conclusions: first, governments should enhance the pertinence and compulsion of their epidemic prevention policies, which should include setting up graded early warning and emergency response mechanisms for different major infectious countries, and using big data and other technologies to collect the customs entry information of various overseas personnel quickly. They should also include monitoring the flow of cross-border personnel to observe the national epidemic in real time and effectively reduce the risk of overseas epidemics affecting. In addition, it is still necessary to establish a punishment mechanism compatible with anti-epidemic policies, which can reduce the risk of the outward spread of COVID-19 in the country. Second, a systematic and efficient risk assessment mechanism for public health emergencies with unified powers and responsibilities should be established; at the same time, adhering to the policy of “putting prevention first,” continuously increasing capital investment for risk assessment, strengthening the construction of specialized teams for risk assessment, and establishing a normalization mechanism for risk assessment are of vital importance. Additionally, the rights and responsibilities of the risk assessment team should be clearly defined in a legal form, and the mechanism arrangement for the risk assessment of public health emergencies should also be established to ensure timely identification of the input risks of overseas epidemics. Finally, countries should prioritize actively guiding public opinion, strengthening the supervision of public opinion, and releasing authentic and reliable information to maintain the stability of the domestic market in the post-epidemic era. Meanwhile, counseling public psychological, avoiding the spread of panic, and being wary of the risk that domestic and foreign capitals exaggerate the COVID-19 epidemic and speculate about the market to undermine domestic economic order cannot be ignored.

This paper discusses the path of the global COVID-19 spread, the degree of the contagion and the transmission tendency of the epidemic. The limitation of our reasoning is the lack of integration of theoretical modeling for the dynamics of epidemiology. Future longitudinal studies are needed to focus on the basic unit of the global COVID-19 epidemic transmission, and the clustering pattern of the contagion.

## Data Availability Statement

Publicly available datasets were analyzed in this study. This data can be found here: https://coronavirus.jhu.edu/data.

## Author Contributions

LX: conceptualization, validation, writing original draft, supervision, and funding acquisition. SM: methodology and writing—review and editing. LY: writing—review and editing and visualization. WW: software, data curation, project administration, and visualization. ZY: resources, formal analysis, and editing. All authors contributed to the article and approved the submitted version.

## Funding

We acknowledge the financial support from Shandong Provincial Natural Science Foundation (Grant Number: ZR2020QG032), Shandong Provincial Social Science Planning Office (Grant Numbers: 21DGLJ12 and 21DJJJ02), Taishan Scholars Program of Shandong Province, China (Grant Numbers: ts201712059 and tsqn201909135) and Youth Innovative Talent Technology Program of Shandong Province, China (Grant Number: 2019RWE004). All errors remain our own.

## Conflict of Interest

The authors declare that the research was conducted in the absence of any commercial or financial relationships that could be construed as a potential conflict of interest.

## Publisher's Note

All claims expressed in this article are solely those of the authors and do not necessarily represent those of their affiliated organizations, or those of the publisher, the editors and the reviewers. Any product that may be evaluated in this article, or claim that may be made by its manufacturer, is not guaranteed or endorsed by the publisher.

## Footnotes

^1^R value is the product of the transmission rate infection-producing contacts per unit time.

^2^The principal eigenvalue of the non-constant disease free equilibrium.

^3^Data source: The World Bank Database.

## References

[1] Chan JFW Yuan S Kok KH To KKW Chu H Yang J. A familial cluster of pneumonia associated with the 2019 novel coronavirus indicating person-to-person transmission: a study of a family cluster. Lancet. (2020) 395:514–23. 10.1016/S0140-6736(20)30154-9 31986261 PMC7159286

[2] Bai P Tang Y Zhang W Zeng M. Does economic policy uncertainty matter for healthcare expenditure in China? a spatial econometric analysis. Front Public Health. (2021) 9:455. 10.3389/fpubh.2021.673778 34017814 PMC8129179

[3] Zhang W Zhang X Tian X Sun F. Economic policy uncertainty nexus with corporate risk-taking: the role of state ownership and corruption expenditure. Pacific Basin Finance J. (2021) 65:101496. 10.1016/j.pacfin.2021.101496

[4] White H Kim TH Manganelli S. VAR for VaR: measuring tail dependence using multivariate regression quantiles. J Econom. (2015) 187:169–88. 10.1016/j.jeconom.2015.02.004

[5] Alarcon Falconi TM Estrella B Sempértegui F Naumova EN. Effects of data aggregation on time series analysis of seasonal infections. Int J Environ Res Public Health. (2020) 17:5887. 10.3390/ijerph17165887 32823719 PMC7460497

[6] Melin P Monica JC Sanchez D Castillo O. Multiple ensemble neural network models with fuzzy response aggregation for predicting COVID-19 time series: the case of Mexico. Healthcare. (2020) 8:181. 10.3390/healthcare8020181 32575622 PMC7349072

[7] Papastefanopoulos V Linardatos P Kotsiantis S. COVID-19: a comparison of time series methods to forecast percentage of active cases per population. Appl Sci. (2020) 10:3880. 10.3390/app10113880

[8] Anastassopoulou C Russo L Tsakris A Siettos C. Data-based analysis, modelling and forecasting of the COVID-19 outbreak. PLoS ONE. (2020) 15:e0230405. 10.1371/journal.pone.0230405 32231374 PMC7108749

[9] Chimmula VKR Zhang L. Time series forecasting of COVID-19 transmission in Canada using LSTM networks. Chaos Solitons Fractals. (2020) 135:109864. 10.1016/j.chaos.2020.109864 32390691 PMC7205623

[10] Vokó Z Pitter JG. The effect of social distance measures on COVID-19 epidemics in Europe: an interrupted time series analysis. Geroscience. (2020) 42:1075–82. 10.1007/s11357-020-00205-0 32529592 PMC7288252

[11] Liu T Hu J Kang M Lin L Zhong H Xiao J. Transmission dynamics of 2019 novel coronavirus (2019-nCoV). (2020). 10.2139/ssrn.3526307

[12] Zhu CC Zhu J. Dynamic analysis of a delayed COVID-19 epidemic with home quarantine in temporal-spatial heterogeneous via global exponential attractor method. Chaos Solitons Fractals. (2021) 143:110546. 10.1016/j.chaos.2020.110546 33519115 PMC7832886

[13] Kermack WO McKendrick AG. A contribution to the mathematical theory of epidemics. Proceedings of The Royal Society Of London Series A, Containing Papers Of A Mathematical And Physical Character. (1927) 115:700–21. 10.1098/rspa.1927.011827300915

[14] Teles P. Predicting the Evolution of COVID-19 In Portugal Using An Adapted SIR Model Previously Used in South Korea for the MERS Outbreak. arXi. (2020). Available online at: https://xueshu.baidu.com/usercenter/paper/show?paperid=1f1k02w0cy4404q0gn4e02p0su578788andsite=xueshu_seandhitarticle=1

[15] Tang B Wang X Li Q Bragazzi NL Tang S Xiao Y. Estimation of the transmission risk of the 2019-nCoV and its implication for public health interventions. J clin med. (2020) 9:462. 10.3390/jcm9020462 32046137 PMC7074281

[16] Pandey G Chaudhary P Gupta R Pal S. SEIR and regression model based COVID-19 outbreak predictions in India. arXiv preprint arXiv. (2020). 10.2196/preprints.1940630181109

[17] Su L Hong N Zhou X He J Ma Y Jiang H. Evaluation of the secondary transmission pattern and epidemic prediction of COVID-19 in the four metropolitan areas of China. Front Med. (2020) 7:171. 10.3389/fmed.2020.00171 32574319 PMC7221060

[18] Huang D Tao H Wu Q Huang SY Xiao Y. Modeling of the long-term epidemic dynamics of COVID-19 in the United States. Int J Environ Res Public Health. (2021) 18:7594. 10.3390/ijerph18147594 34300045 PMC8305610

[19] Zhao Z Li X Liu F Zhu G Ma C Wang L. Prediction of the COVID-19 spread in African countries and implications for prevention and control: a case study in South Africa, Egypt, Algeria, Nigeria, Senegal and Kenya. Sci Total Environ. (2020) 729:138959. 10.1016/j.scitotenv.2020.138959 32375067 PMC7182531

[20] Yang Z Zeng Z Wang K Wong SS Liang W Zanin M. Modified SEIR and AI prediction of the epidemics trend of COVID-19 in China under public health interventions. J Thorac Dis. (2020) 12:165. 10.21037/jtd.2020.02.64 32274081 PMC7139011

[21] He S Peng Y Sun K. SEIR modeling of the COVID-19 and its dynamics. Nonlinear Dyn. (2020) 101:1667–80. 10.1007/s11071-020-05743-y 32836803 PMC7301771

[22] Jiang YX Xiong X Zhang S Wang JX Li JC Du L. Modeling and prediction of the transmission dynamics of COVID-19 based on the SINDy-LM method[J]. Nonlinear Dyn. (2021) 105:2775–94. 10.1007/s11071-021-06707-6 34312574 PMC8295551

[23] Kamra N Zhang Y Rambhatla S Meng C Liu Y. PolSIRD: Modeling Epidemic spread under intervention policies. J Healthc Inform Res. (2021) 5:231–48. 10.1007/s41666-021-00099-3 34151134 PMC8202228

[24] Zou D Wang L Xu P Chen J Zhang W Gu Q. Epidemic model guided machine learning for COVID-19 forecasts in the United States. medRxiv. (2020). 10.1101/2020.05.24.20111989

[25] James N Menzies M. Cluster-based dual evolution for multivariate time series: Analyzing COVID-19. Chaos. (2020) 30:061108. 10.1063/5.0013156 32611104 PMC7328914

[26] Park JY. Spatial visualization of cluster-specific Covid-19 transmission network in South Korea during the early epidemic phase. medRxiv. (2020). 10.1101/2020.03.18.20038638

[27] Yum S. Social network analysis for coronavirus (COVID-19) in the United States. Soc Sci Q. (2020) 101:1642–7. 10.1111/ssqu.12808 32836475 PMC7283848

[28] Jo W Chang D You M Ghim GH. A social network analysis of the spread of COVID-19 in South Korea and policy implications. Sci Rep. (2021) 11:1–10. 10.1038/s41598-021-87837-0 33883601 PMC8060276

[29] Wang P Lu J Jin Y Zhu M Wang L Chen S. Statistical and network analysis of 1212 COVID-19 patients in Henan, China. Int J Infect Dis. (2020) 95:391–8. 10.1016/j.ijid.2020.04.051 32339715 PMC7180361

[30] Skums P Kirpich A Baykal PI Zelikovsky A Chowell G. Global transmission network of SARS-CoV-2: From outbreak to pandemic. MedRxiv. (2020). 10.1101/2020.03.22.20041145 32511620 PMC7276047

[31] Antonakakis N Gabauer D Gupta R Plakandaras V. Dynamic connectedness of uncertainty across developed economies: A time-varying approach. Econ Lett. (2018) 166:63–75. 10.1016/j.econlet.2018.02.011

[32] Antonakakis N Chatziantoniou I Gabauer D. Refined measures of dynamic connectedness based on time-varying parameter vector autoregressions. J Risk Financ Manag. (2020) 13:84. 10.3390/jrfm13040084

[33] Gabauer D Gupta R. On the transmission mechanism of country-specific and international economic uncertainty spillovers: evidence from a TVP-VAR connectedness decomposition approach. Econ Lett. (2018) 171:63–71. 10.1016/j.econlet.2018.07.007

[34] Gabauer D Gupta R. Spillovers across macroeconomic, financial and real estate uncertainties: a time-varying approach. Structural Change Economic Dynamics. (2020) 52:167–73. 10.1016/j.strueco.2019.09.009

[35] Diebold FX Yilmaz K. Measuring financial asset return and volatility spillovers, with application to global equity markets. Econ J. (2009) 119:158–71. 10.1111/j.1468-0297.2008.02208.x

[36] Diebold FX Yilmaz K. Better to give than to receive: predictive directional measurement of volatility spillovers. Int J Forecast. (2012) 28:57–66. 10.1016/j.ijforecast.2011.02.00634745369

[37] Diebold FX Yilmaz K. On the network topology of variance decompositions: Measuring the connectedness of financial firms. J Econometrics. (2014) 182:119–134. 10.1016/j.jeconom.2014.04.01234745369

[38] Jebabli I Arouri M Teulon F. On the effects of world stock market and oil price shocks on food prices: an empirical investigation based on TVP-VAR models with stochastic volatility. Energy Econ. (2014) 45:66–98. 10.1016/j.eneco.2014.06.008

[39] Antonakakis N Gabauer D. Refined Measures Of Dynamic Connectedness Based on TVP-VAR. (2017). Available online at: https://mpra.ub.uni-muenchen.de/id/eprint/78282

[40] Liu X Yin K Cao Y. Contribution of the optimization of financial structure to the real economy: evidence from china's financial system using TVP-VAR model. Mathematics. (2021) 9:2232. 10.3390/math9182232

[41] Koop G Pesaran MH Potter SM. Impluse response analysis in nonlinear multivariate models. J Econom. (1996) 74:119–47. 10.1016/0304-4076(95)01753-4

[42] Pesaran HH Shin Y. Generalized impulse response analysis in linear multivariate models. Econ Lett. (1998) 58:17–29. 10.1016/S0165-1765(97)00214-011369086

[43] Fan C Cai T Gai Z Wu Y. The relationship between the migrant population's migration network and the risk of COVID-19 transmission in China—empirical analysis and prediction in prefecture-level cities. Int J Environ Res Public Health. (2020) 17:2630. 10.3390/ijerph17082630 32290445 PMC7215340

[44] Zhang Y Hamori S. Do news sentiment and the economic uncertainty caused by public health events impact macroeconomic indicators? evidence from a TVP-VAR decomposition approach. Q Rev Econ Finance. (2021) 82:145–62. 10.1016/j.qref.2021.08.003

