# Supplementary material for: Modeling the Global Dynamic Contagion of COVID-19
Source: Front Public Health. 2022 Jan 14;9:809987. doi: 10.3389/fpubh.2021.809987 (PMC8795671; doi:10.3389/fpubh.2021.809987)
Supplement: Supplementary file 1 [file Data_Sheet_1.docx]

Appendix: Additional results





Figure A1: The dynamic net pairwise connectedness of the COVID-19 epidemic (Part one)

Notes: The results are based on the connectedness approach based on TVP-VAR model，and the solid line represents the change trend of the net COVID-19 contagion from one country to another.





Figure A2: The dynamic net pairwise connectedness of the COVID-19 epidemic (Part two)

Notes: The results are based on the connectedness approach based on TVP-VAR model，and the solid line represents the change trend of the net COVID-19 contagion from one country to another.





Figure A3: The dynamic net pairwise connectedness of the COVID-19 epidemic (Part three)

Notes: The results are based on the connectedness approach based on TVP-VAR model，and the solid line represents the change trend of the net COVID-19 contagion from one country to another.





Figure A4: The dynamic net pairwise connectedness of the COVID-19 epidemic (Part four)

Notes: The results are based on the connectedness approach based on TVP-VAR model，and the solid line represents the change trend of the net COVID-19 contagion from one country to another.





Figure A5: The dynamic net pairwise connectedness of the COVID-19 epidemic (Part five)

Notes: The results are based on the connectedness approach based on TVP-VAR model，and the solid line represents the change trend of the net COVID-19 contagion from one country to another.





Figure A6: The dynamic net pairwise connectedness of the COVID-19 epidemic (Part six)

Notes: The results are based on the connectedness approach based on TVP-VAR model，and the solid line represents the change trend of the net COVID-19 contagion from one country to another.





Figure A7: The dynamic net pairwise connectedness of the COVID-19 epidemic (Part seven)

Notes: The results are based on the connectedness approach based on TVP-VAR model，and the solid line represents the change trend of the net COVID-19 contagion from one country to another.





Figure A8: The dynamic net pairwise connectedness of the COVID-19 epidemic (Part eight)

Notes: The results are based on the connectedness approach based on TVP-VAR model，and the solid line represents the change trend of the net COVID-19 contagion from one country to another.
